# The NS1 protein of influenza a virus interacts with heat shock protein Hsp90 in human alveolar basal epithelial cells: Implication for virus-induced apoptosis

**DOI:** 10.1186/1743-422X-8-181

**Published:** 2011-04-19

**Authors:** Chuanfu Zhang, Yutao Yang, Xiaowei Zhou, Zhixin Yang, Xuelin Liu, Zhiliang Cao, Hongbin Song, Yuxian He, Peitang Huang

**Affiliations:** 1 Institute of Biotechnology, Academy of Military Medical Sciences, Beijing 100071, P.R.China; 2 Institute of Disease Control and Prevention, Chinese Academy of Military Medical Sciences, Beijing, P.R.China; 3 Institute of Pathogen Biology, Chinese Academy of Medical Sciences and Peking Union Medical College, Beijing 100730, P.R.Chiina; 4 Beijing Institute for Neuroscience, Capital Medical University, Beijing 100069, China

## Abstract

**Background:**

Our previous study showed that the NS1 protein of highly pathogenic avian influenza A virus H5N1 induced caspase-dependent apoptosis in human alveolar basal epithelial cells (A549), supporting its function as a proapoptotic factor during viral infection, but the mechanism is still unknown.

**Results:**

To characterize the mechanism of NS1-induced apoptosis, we used a two-hybrid system to isolate the potential NS1-interacting partners in A549 cells. We found that heat shock protein 90 (Hsp90) was able to interact with the NS1 proteins derived from both H5N1 and H3N2 viruses, which was verified by co-immunoprecitation assays. Significantly, the NS1 expression in the A549 cells dramatically weakened the interaction between Apaf-1 and Hsp90 but enhanced its interaction with cytochrome c (Cyt c), suggesting that the competitive binding of NS1 to Hsp90 might promote the Apaf-1 to associate with Cyt c and thus facilitate the activation of caspase 9 and caspase 3.

**Conclusions:**

The present results demonstrate that NS1 protein of Influenza A Virus interacts with heat hock protein Hsp90 and meidates the apoptosis induced by influenza A virus through the caspase cascade.

## Background

Influenza A virus is a globally important human and animal respiratory pathogen responsible for both seasonal influenza outbreaks and periodic world-wide pandemics. Its genome contains eight segmented and negative-stranded RNAs encoding a total of eleven proteins (HA, NA, NP, M1, M2, NS1, NEP, PA, PB1, PB1-F2, PB2). The NS1 is a 26,000 dalton non-structural protein expressed only within the infected cells. It is accumulated in the cell nucleus at early times during infection and can be presented in the cytoplasm at latter times. Previous studies have demonstrated that the NS1 protein is an important molecular determinant of virulence factor and contributes significantly disease pathogenesis by modulating a number of virus and host-cell processes [1-4]. For example, it can inhibit nuclear export of polyadenylated mRNAs, bind to small nuclear RNA (snRNA) and block pre-mRNA splicing, and suppress the interferon response in the virus-infected cell leading to unimpaired virus production.

Many virus infections induce apoptosis of host cells while some viruses have evolved mechanisms to inhibit apoptotic events. It has been demonstrated that influenza A viruses can result in apoptosis in numerous cell types, both in vivo [5-7] and in vitro [8-16], but the mechanism of virus-induced apoptosis is not well known. Several viral factors, including neuraminidase, M1, NS1, NA and PB1-F2, from different strains of human influenza viruses have been reported to be related apoptosis induction [17-22]. The NS1 protein of influenza A viruses was shown to induce apoptosis in human cells [13,23], but some observations, in a sharp contrast, suggested its role in inhibiting apoptosis [11,21]. Obviously, these contradictory results might be caused by the differences of virus subtypes and strains, as well as the host cell systems being used, highlighting further investigations are needed to clarify whether the NS1 protein is a proapoptotic or antiapoptotic factor in infected cells [24]. We have recently showed that the NS1 protein of highly pathogenic avian influenza A virus H5N1 could induce caspase-dependent apoptosis in human alveolar basal epithelial cells (A549), supporting its function as a proapoptotic factor during viral infection [25]. In this study, our main purpose was to characterize the molecular mechanism of NS1-induced apoptosis. First, we confirmed further that the NS1 protein is a strong inducer of apoptosis by using an H3N2 strain. With a two-hybrid system and co-immunoprecitation assays we identified the heat shock protein 90 (Hsp90) as a binding partner for the NS1 protein of both H5N1 and H3N2 strains. Furthermore, our data suggested that the NS1-Hsp90 interaction might competitively promote the association of Apaf-1 with Cyt c and thus activate the caspase cascade.

## Materials and methods

### Viruses and cells

Influenza A/chicken/Jilin/2003(H5N1)and A/swine/Colorado/1/1977(H3N2)viruses were grown in the allantoic cavities of 10-day-old embryonated chicken eggs. A549 cells were passaged in Dulbecco's modified Eagle's tissue culture medium (DMEM) containing 10% fetal calf serum at 37°C in a 5% CO_2 _incubator. The confluent cell monolayers grown in 25-mm dishes were lysed in immunoprecipitation assay buffer containing 150 mM NaCl, 1.0% Nonidet P-40 (NP-40), 0.5% deoxycholate, 0.1% sodium dodecyl sulfate (SDS), and 50 mM Tris-HCl (pH 8.0). The cell lysates were clarified by centrifugation for 10 min at 13,000 × *g*, and the supernatants were used for immunoblot analysis.

### Construction of expression plasmids

Total RNA was extracted from the cell lysate using the QIAamp viral RNA mini kit (Qiagen, Hilden, Germany). The full-length NS1 gene from H5N1 or H3N2 were amplified using the SuperScript III one-step reverse transcription-PCR (RT-PCR) system with Platinum *Taq *high-fidelity polymerase (Invitrogen, Carlsbad, CA) and ligated into pSos vector according to the protocol of the vector manufacturer (Invitrogen). Clones were screened by PCR and sequencing. Competent *Escherichia coli *DH5α cells were transformed with the plasmids, and the plasmids were amplified and purified using a high-purity plasmid purification kit (Qiagen). The constructions of expression plasmids, including pCMV-myc/NS1 (H5N1 or H3N2), pCMV-Flag/Hsp90, pSos/NS1 (H5N1 or H3N2), pBIND/NS1 (H5N1 or H3N2), pACT/Hsp90 followed standard cloning procedures. 5NS1 denotes the NS1 derived from H5N1, 3NS1 denotes the NS1 from H3N2.

### Electron microscopic analysis

A549 cells were transfected with pCMV-myc/3NS1 by using Lipofectamine 2000 reagent (Invitrogen). After 24 h, cells were collected, digested, washed with phosphate buffered solution (PBS), fixed with 4% glutaraldehyde for 2 h, and then fixed with osmium tetroxide for 1 h, stained with uranium acetate, embedded into epoxide resin. After sectioning into ultra-thin slices, the cells were stained with lead citrate and examined under transmission electron microscopy (TECNAI10).

### Flow cytometric analysis

To determine the apoptosis rate, an Annexin V-FITC apoptosis detection kit (BD Pharmingen, San Diego, CA) was used to detect early apoptotic activity according to the manufacturer's instruction, with slight modifications. After 24 h transfected as described above, A549 cells were harvested and washed twice with ice-cold PBS and resuspended in 100 ml of binding buffer. A total of 5 ml of Annexin V-FITC and 10 ml of propidium iodide (PI) were added and the mixture was incubated for 30 min in the dark. Finally, the binding buffer was added to the cells and the mixture was analyzed with a Flow cytometer (Becton Dickinson Co., San Jose, CA), using an FITC signal detector (FL1) for Annexin V staining and a phycoerythrin emission signal detector for PI staining. The apoptotic percentage of 10,000 cells was determined. All the experiments reported in this study were performed three times. The data were analyzed by using WinMDI 2.8 software (Scripps Institute, La Jolla, CA) for calculation of percentage cells with apoptosis per group.

### Expression of caspase-9 and caspase-3

The expression of caspase-9 and caspase-3 in the NS1-transfected A549 cells was measured by Western-blot analysis. Briefly, the monolayer of cells transfected with pCMV-myc/3NS1 were lysed with ice-cold lysis buffer (150 mM Tris-HCl, pH 8.0, 50 mM NaCl, 1 mM EDTA, 0.5% Nonidet P-40, 1 tablet Complete Mini protein inhibitor mixture/10 ml (Roche Applied Science, Indianapolis, IN) and 0.7 μg/ml pepstatin), and the cell lysates were clarified by centrifugation at 20,000 *g *for 10 min at 4°C. The caspase-9 and caspase-3 activities were determined according to the supplemental protocols of the Caspase-9/Mch6 Colorimetric Assay kit and Caspase-3/CPP32 Colorimetric Protease Assay kit (MBL, Nagoya, Japan), respectively. Substrate cleavage, which resulted in the release of pNA (405nm), was measured using a Multiskan Ascent plate reader.

### Isolation of NS1-binding partners by CytoTrap two-hybrid system

The CytoTrap two-hybrid system (Stratagene, La Jolla, CA) was used to screen a human fetal lung plasmid cDNA library which has 5.3 × 10^6 ^primary colonies and an average insert size of 1.2 kb according to the manufacturer's instructions (Stratagene). The NS1 gene of H5N1 or H3N2 was fused to the N-terminal 1070 residues of human Sos and used as a bait. The prey cDNAs were fused to the myristoylation signal of v-Src that anchors the fusion proteins to the plasma membrane. The cdc25H was firstly co-transformed with pSos-5NS1 or pSos-3NS1 together with pYES-mGAP to reduce isolation of Ras GTPase false positive clones. The pretransformed cdc25H was then transformed with 3 μg of pMyr-cDNA library plasmids for the CytoTrap screening. The resulting transformants were grown for 5 days at 22°C on selective minimal glucose plates (complete supplement mixture-LEU-URA-TRP). After plating the replica onto the selective minimal galactose plates, the colonies that showed galactose-dependent growth under restrictive conditions were selected for plasmid preparation. The isolated plasmid was then transformed into *Escherichia coli *DH5α and selected on 50 ng/μl chloramphenicol for the presence of the cDNA insert-containing pMyr plasmid. The protein interactions of putatively positive colonies were confirmed by retransformation of the cdc25H yeast strain with both the cDNA-containing pMyr plasmid and pSos-5NS1 or pSos-3NS1. The empty pSos was used as a negative control. Only those clones growing on galactose media at the restrictive temperature of 37°C after 6 days were defined as true positives. Co-transformation of cdc25H cells with pSos-MafB and pMyr-target cDNA, pSos and pMyr-target cDNA, pSos-MafB and pMyr-Lamin C, pSos and pMyr, pSos-5NS1and pMyr-Lamin C, and pSos-5NS1 and pMyr served as negative controls, whereas co-transformation of cdc25H cells with pSos-MafB and pMyr-MafB served as a positive control.

### Mammalian two-hybrid system

The CheckMate mammalian two-hybrid system (Promega, Madison, WI) was used to characterize the protein interactions in the mammalian cells. In this system, the pBind vector contains the yeast GAL4 DNA-binding domain upstream of a multiple cloning region. The pACT vector contains the herpes simplex virus VP16 activation domain upstream of a multiple coding region. The NS1 and Hsp90 genes were cloned into the pBind and pACT vectors to generate fusion proteins with the GAL4 DNA-binding domain and the VP16 activation domain, respectively. The pG5luc vector contains five GAL4 binding sites upstream of a minimal TATA box, which in turn is upstream of the firefly luciferase gene (luc+). The pGAL4 and pVP16 fusion constructs were transfected along with the pG5luc vector into mammalian cells. The plasmids were co-transfected using Lipofectamine 2000 reagent (Invitrogen) according to the manufacturer's protocol in the following combinations: 1) pACT vector + pBind vector + pG5*luc *vector; 2) pACT vector + pBind-5NS1(or -3NS1) vector + pG5*luc *vector; 3) pACT-Hsp90 vector **+ **pBind vector + pG5*luc *vector; 4) pACT-Hsp90 vector + pBind-5NS1(or -3NS1) vector + pG5*luc *vector; 5) pACT-MyoD + pBind-Id + pG5*luc *vector; 6) blank control. After transfection 24h, the cells were lysed and the luciferase activity was quantitated according to the method described previously (Miranda et al., 1993). Briefly, 10 μl cell lysates were added to 100 μl 1mM ATP (Sigma, Buchs, Switzerland)/10mM MgAc/0.1mg/ml BSA in 250mM Tris-HCl, pH 7.5. Samples were injected with 100 μl 200 μg coenzyme A/30 μg luciferine (Sigma)/ml in 12.5mM PIPES, pH 6.5 and light emission was measured after a delay of 0.3s during a 10s interval in a MicroLumatPlus luminometer (Berthold Technologies, Bad Wildbach, Germany). All experiments were performed at least three times and the samples were measured in duplicates.

### Co-immunoprecipitation assays

To study the interaction between NS1 protein and Hsp90, 1.2 × 10^6 ^A549 cells cultured in DMEM containing 10% fetal calf serum were cotransfected with pCMV-Flag/Hsp90 and pCMV-myc/5NS1 or pCMV-myc/3NS1 using Lipofectamine 2000 reagent (Invitrogen) according to the manufacturer's protocol. After a 24 h incubation, cells were lysed with ice-cold lysis buffer (150mM Tris-HCl, pH 8.0, 50mM NaCl, 1 mM EDTA, 0.5% Nonidet P-40, 1 tablet Complete Mini protein inhibitor mixture/10 ml (Roche Applied Science), and 0.7 μg/ml pepstatin), and the lysates were clarified by centrifugation at 20,000 *g *for 10 min at 4°C. Supernatants were applied to 50 μl of anti-Myc agarose or anti-Flag agarose (Sigma) respectively and incubated overnight at 4°C. Subsequently, the immunoprecipitates were washed three times with washing buffer (50mM Tris-HCl, pH 7.5, 250mM NaCl) and subjected together with total lysates to SDS-PAGE and immunoblot analysis. Proteins were detected using HRP-conjugated mouse monoclonal anti-Flag antibody (1:3500) and anti-Myc antibody (1:4000; Sigma) or rabbit anti-NS1 serum (1:1000) followed by incubation with HRP-conjugated anti-rabbit antibody (1:5000; Amersham Biosciences). To analyze whether the NS1 expression affects the interaction between Hsp90 and Apaf-1 or between Cyt c and Apaf-1, the co-immunoprecitation assays were similarly performed. Briefly, the A549 cells were transfected with the plasmids expressing the NS1 proteins as described above, the cell supernatants were prepared and applied to anti-Hsp90 agarose or anti-Cyt c agarose. The immunoprecipitates were detected by immunoblotting with HRP-conjugated goat anti-Apaf-1 antibody.

## Results

### Expression of NS1 protein induced apoptosis in A549 cells

**Figure 1:**
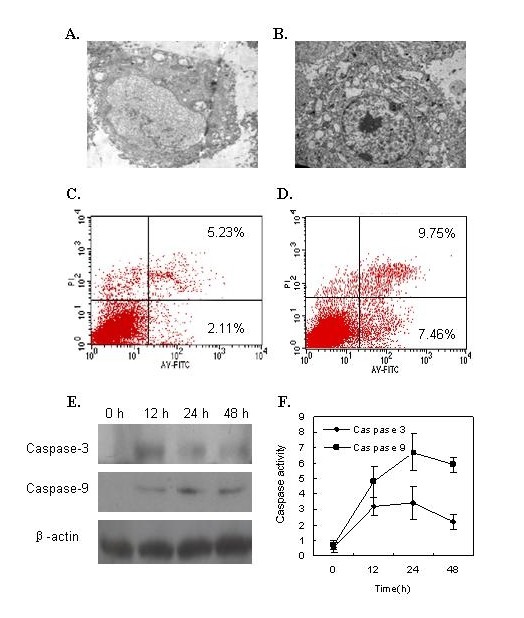
**Influenza A virus NS1 protein induces apoptosis in A549 cell**. The transmission electron microscopy reveals that the cells transfected with pCMV-Myc empty vector shows an intact nuclear shape (A) and transfected with pCMV-Myc/3NS1 shows condensed, shrunk chromatins aggregated along inside the nuclear membrane, and apoptotic bodies (B). The flow cytometric analysis of A549 cells transfected with the empty pCMV-Myc vector (C) or transfected with pCMV-Myc/3NS1 (D). The dot plot diagrams represent typical apoptotic and necrotic cell populations detected by Annexin V-FITC and PI staining. The lower left quadrants of the panels show viable intact cells, which were negative for Annexin V-FITC binding and excluded PI staining (FITC^-^/PI^-^); the upper right quadrants show nonviable, necrotic cells, which were positive for Annexin V-FITC binding and PI uptake (FITC^+^/PI^+^). The lower right quadrants represent apoptotic cells, positive for Annexin V-FITC and negative for PI (FITC^+^/PI^-^). The expression of caspase 3 and caspase 9 posttransfection 0 h, 12 h, 24 h, and 48 h were detected by Western blotting (E), and the enzyme activities of caspase-3 and caspase-9 in the NS1-transfected A549 cells were detected by Caspase-3/CPP32 Colorimetric Protease Assay and Caspase-9/Mch6 Colorimetric Assay, respectively (F).

We have recently reported that the NS1 protein of H5N1 could induce caspase-dependent apoptosis in A549 cells. Here, we sought to clarify whether the NS1 of influenza A/swine/Colorado/1/1997, a H3N2 strain, could induce apoptosis. After the plasmid pCMV-myc/3NS1 was transfected into A549 cells, we examined the morphological and ultrastructural features of the transfected cells. Figure 1A shows the empty vector-transfected cells with normal silhouettes, but the NS1-transfected cells appear to be characteristics of apoptotic cells, including nuclear condensation, chromatin aggregation to the nuclear membrane and the apoptotic bodies in some cells (Figure 1B). The NS1-transfected A549 cells were stained with annexin V-FITC and PI and analyzed by flow cytometry. Compared to the empty vector-transfected cells (Figure 1C shows 2.11% Annexin V^+^/PI^- ^and 5.23% Annexin V^+^/PI^+ ^), the NS1-transfected cells were 7.46% Annexin V^+^/PI^- ^(early apoptosis) and 9.75% Annexin V^+^/PI^+ ^(latter apoptosis).

We then investigated whether the caspase pathways were involved in the apoptosis induced by H3N2 NS1 proteins. The A549 cells were transfected with the NS1-expressing plasmid and the cell lysates were prepared. The expression of caspase-9 and caspase-3 was detected by Western-blotting. As shown in Figure 1E, both apoptosis-related caspase-9 and caspase-3 were activated in the transfected A549 cells. The active fragments of caspase-9 and caspase-3 could be detected at post-transfection 12, 24, and 48 hrs. The enzyme activities of caspase-9 and caspase-3 were further measured and the results showed that both apoptotic enzymes were activated in the NS1-transfected A549 cells (Figure 1F). These data confirmed that the NS1 protein of influenza A virus H3N2, like that of H5N1, induced caspase-dependent apoptosis in the transfected A549 cells.

### Identification of Hsp90 as a NS1-binding partner

**Figure 2:**
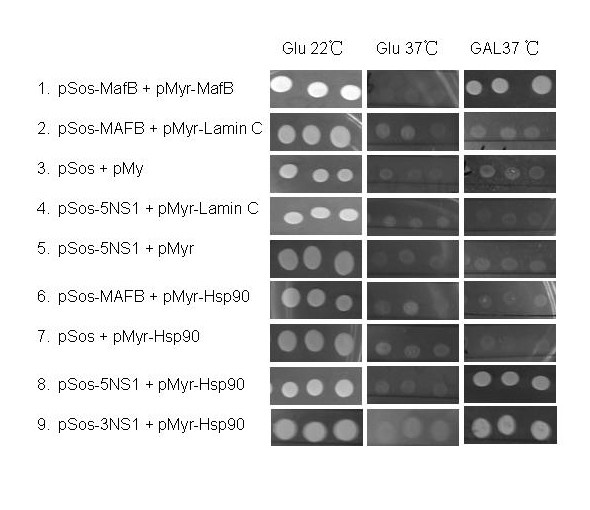
**Identification of NS1-Hsp90 interaction *in yeast *by the CytoTrap two-hybrid system**. Various bait (pSos) and prey (pMyr) plasmids were used for co-transformation of cdc25H yeast cells, representing a positive control (*lane 1*) and negative controls (*lanes 2-7*). Five independent cdc25H transformants were spotted on glucose (*GLU*) medium at 22°C (*left panel*) or 37°C (*middle panel*) and on galactose (*GAL*) medium at 37°C (*right panel*). Yeast transformants expressing NS1 (H5N1 and H3N2) and Hsp90 grew efficiently on galactose medium at 37°C (*right panel, lane 8*). 5NS1 represents H5N1 NS1 and 3NS1 represents H3N2 NS1.

To explore the mechanism of influenza A virus NS1 protein-induced apoptosis, we used the CytoTrap two-hybrid system to isolate the potential NS1-interacting proteins. This system was developed to greatly increase the opportunities for identifying unique protein-protein interactions by taking the search to the yeast cytoplasm. Unlike in the nucleus, the proteins expressed in the cytoplasm may undergo posttranslational modifications. The coding region of H5N1 or H3N2 NS1 gene was used in the CytoTrap screen. The yeast cells expressing the Sos-NS1 were transformed with a human lung cDNA library. The prey was expressed as a fusion protein with a myristylation sequence that anchors the fusion protein onto the plasma membrane. If the bait protein physically interacted with the prey protein, Sos was recruited to the membrane, where activates the yeast RAS-signaling pathway and allows cdc25Hα to grow at 37°C. A total of 24 colonies of ~2.2 million transformants showed galactose-dependent growth under restrictive conditions. Only those clones showing NS1 dependent growth were defined as true positives. One clone was identified to encode an apoptosis-related protein, Hsp90 (Figure 2). Further experiments confirmed that Hsp90 was a target protein of the NS1 bait derived from both H5N1 and H3N2.

### Characterization of NS1 and Hsp90 interaction

**Figure 3:**
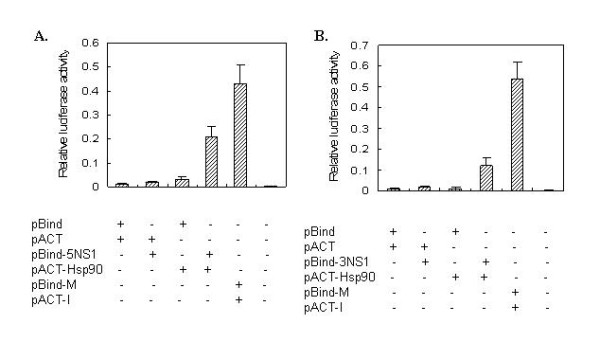
**Characterization of the NS1-Hsp90 interaction in A549 cells by mammalian two-hybrid system**. pACT Vector, pBIND Vector, pBIND-5NS1(or -3NS1) Vector, pACT-Hsp90 and the indicated expression plasmids were co-transfected A549 cells as groping of described above. The values represent the average from three independent experiments for each group.

First, we characterized the interaction between the NS1 and hsp90 by using a mammalian two-hybrid system. The NS1 (H5N1 or H3N2) and Hsp90 genes were cloned into the pBIND and pACT vectors to generate fusion proteins with the GAL4 DNA-binding domain (pBind-NS1) and the VP16 activation domain (pACT-Hsp90), respectively. The pBind-NS1 and pACT-Hsp90 were cotransfected along with the pG5luc vector into A549 cells. The interaction of the two clock components reconstitutes a functional transcription factor that activates a luciferase reporter under the control of a GAL4-based promoter. For GAL4-NS1 of H5N1/Hsp90-VP16, several-folds increase in luciferase activity was observed (Figure 3A). Similar values were also obtained for GAL4-NS1 of H3N2/Hsp90-VP16 (Figure 3B). However, only a minimal activation of the reporter was seen when GAL4-NS1 of H5N1 or H3N2 was expressed alone or together with an unrelated fusion protein (VP16) or when the VP16 fusions were co-transfected with the Gal4 DBD alone. These results confirmed that the NS1 protein can interact with Hsp90 in A549 cells.

**Figure 4:**
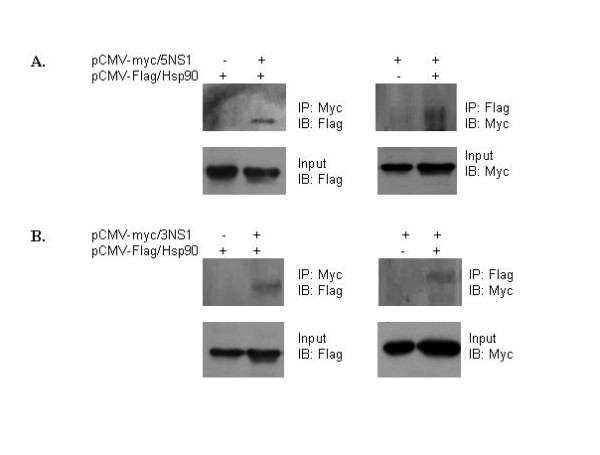
**Characterization of the NS1-Hsp90 interaction in A549 cells by Coimmunoprecipitation**. A549 cells were co-transfected with the plasmids encoding Myc-tagged NS1 (A. H5N1; B. H3N2) and Flag -tagged Hsp90. Cell lysates were immunoprecipitated with anti-Myc antibody to capture NS1 and coimmunoprecipitated Hsp90 was detected by immunoblotting with anti-Hsp90 antibody. Lysates were prepared as in described above, but immunoprecipitated with anti Flag antibody to capture Hsp90, and the associated NS1 of H5N1 was detected by immunoblotting with anti-Myc antibody. (5NS1 represent NS1 of influenza H5N1; 3NS1 represent NS1 of influenza H3N2).

We then performed co-immunoprecipitation experiments to further verify the interaction between the NS1 (H5N1 or H3N2) and Hsp90 in A549 cells. The Myc-tagged NS1and pCMV-Flag/Hsp90 were co-expressed in A549 cells. The lysates of the transfected cells were analyzed. First, NS1 protein was precipitated with polyclonal anti-Myc antibodies and Hsp90 was detected by immunoblotting with polyclonal anti- Flag antibodies. It was found that the Hsp90 could be detected in the anti-Myc immunoprecipitates. Simultaneously, the Hsp90 was precipitated from the cell lysates with anti-Flag and the NS1 was detected with anti-Myc (Figure 4A and Figure 4B). Consistently, the NS1 and Hsp90 proteins could be co-immunoprecipitated. These results demonstrated that Hsp90 was a binding partner of influenza NS1 proteins derived from both H5N1 and H3N2.

### The NS1 expression weakened the interaction of Apaf-1 with Hsp90

**Figure 5:**
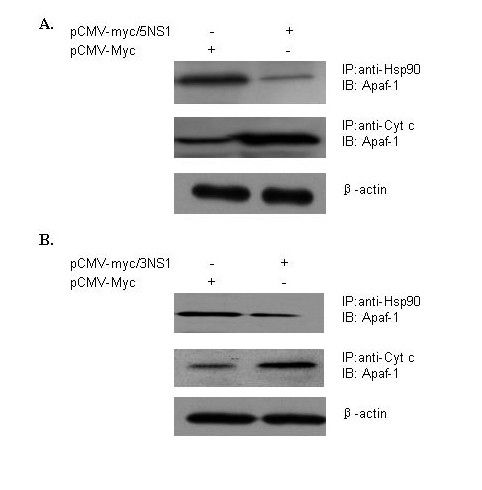
**The NS1 expression affects the interaction of Apaf-1 with hsp90 or Cyt c**. The plasmids expressing H5N1 NS1 (pCMV-myc/5NS1) or H3N2 NS1 (pCMV-myc/3NS1) were transfected into cells for 24 hours and the cell lysates were incubated with anti-Hsp90 antibodies or anti-Cyt c antibodies. The resulting immunoprecipitates were analyzed by immunoblotting with anti-Apaf-1 antibodies.

It is known that Hsp90 can inhibit caspase-depenent apoptosis by its interaction with Apaf-1. We hypothesized that the NS1 expression might compete with Apaf-1 for Hsp90 binding and thus promote the activation of caspases through its interaction with Cyt c. To this end, we applied the co-immunoprecipitation assays to investigate whether the NS1 could affect Apaf-1 in the A549 cells. The plasmids expressing H5N1 NS1 (pCMV-myc/5NS1) or H3N2 NS1 (pCMV-myc/3NS1) were transfected into the cells for 24 hours and the cell lysates were incubated with anti-Hsp90 antibodies or anti-Cyt c antibodies. The resulting immunoprecipitates were analyzed by immunoblotting with anti-Apaf-1 antibodies. As shown in Figure 5, the quantity of Apaf-1 bound to Hsp90 was dramatically decreased in the NS1-transfected cells, whereas the quantity of Apaf-1 bound to Cyt c dramatically increased. As a control, the expression of β-actin had no changes in the NS1-transfected cells compared to the empty vector-transfected cells. These data suggested that the competitive interaction between NS1 and Hsp90 might promote the binding of Apaf-1 to Cyt c, implying a novel pathway for the NS1-mediated caspase cascade during the apoptosis induced by influenza A virus.

## Discussion

In this study, we continued our studies to characterize the functionality and mechanism of influenza A virus NS1 protein in the virus-induced apoptosis. The NS1 gene was cloned from the influenza A/swine/Colorado/1/1997 (H3N2) and expressed in the A549 cells. Consistent with our previous studies from the NS1 protein of highly pathogenic avian influenza A virus H5N1 [25], the H3N2 NS1 protein was also capable to cause apoptotic responses and two major apoptosis-related enzymes, caspase-9 and caspase-3, were significantly activated in the transfected A549 cells. We were attracted to know the signal pathway of NS-1-induced caspase-dependent apoptosis. In an effort to search for the possible interacting partners of the NS1 protein in the A549 cells, we decided to use the CytoTrap two-hybrid system. This approach was designed to isolate the binding partner in the yeast cytoplasm rather than in the nucleus and provide a novel method for detecting protein-protein interactions in vivo. To our knowledge, it had not been used to identify the NS1-interacting proteins. In addition, the use of A549 cells might also provide more chance to find novel targets. In deed, a number of binding partners were hooked out from the human lung cDNA library. Among them we focused on the heat shock protein Hsp90 because it was the only known protein involved in the apoptosis. Consistently, the physiological protein-protein interaction between the NS1 protein and Hsp90 was verified by the mammalian two-hybrid system and co-immunoprecipitation assays.

Similar to other heat shock proteins, Hsp90 is a constitutively abundant molecular chaperone that controls the conformation, stability, activation, intracellular distribution, and turnover of numerous client proteins and involved in cell growth, differentiation, and survival [26,27]. The cytoprotective function of Hsp90 is largely explained by its anti-apoptotic effect. It has been shown that overexpression of Hsp90 can prevent apoptosis triggered by various stimuli [28,29], whereas downregulation or inhibition of its expression is enough to sensitize cells to apoptosis [30-32]. Recently, there is ample evidence to show that Hsp90 plays critical roles in viral replication, and that inhibitors for Hsp90 can impair the growth of many viruses [33-36]. In the case of influenza A virus, Hsp90 was shown to play a role in the nuclear import and assembly of viral RNA polymerase complex by binding to the PB1 and PB2 subunits, and Hsp90 inhibitors geldanamycin and its derivative 17-AAC could inhibit viral growth at early time points [33,37]. Therefore, the identification of Hsp90 as a NS1 protein interacting partner has provided a new clue for exploring the mechanism of influenza virus-induced apoptosis. Structurally, Hsp90 contains three functional domains: the ATP binding domain near the N-terminus, protein binding domain located towards the C-terminus of the amino sequence, and dimerizing domain [38]; the multifunctional protein NS1 contains the N-terminal RNA-binding domain and the C-terminal effector domian and participates in both protein-protein and protein-RNA interactions [39]. How these two molecules physically contact in the cells remains to be further characterized. The molecular motifs responsible for the interaction can help to define their functions in the apoptotic induction.

**Figure 6:**
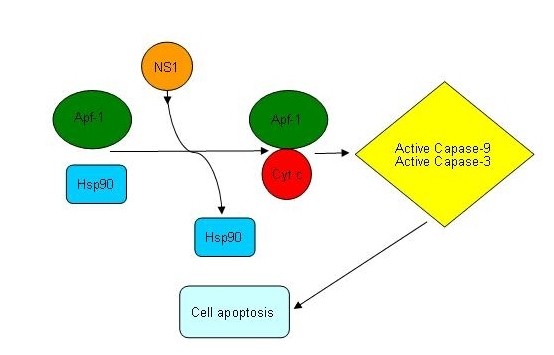
**A proposed model for the molecular mechanism of NS1-induced apoptosis**. Binding of NS1 protein to Hsp90 might compete off the Apaf-1, resulting in the oligomerization of Apaf-1 which promotes the interaction between Apaf-1 and Cyt c and lead to the activation of caspase 9 and caspase 3 and thus the cell apoptosis occur.

Previous studies demonstrated that Hsp90 can interact with key client proteins of the apoptotic pathways including a number of signaling proteins like ligand-depentent transcription factors and signal transducing kinases [26,27,40-42]. Most importantly, Hsp90 can associate directly with Apaf-1 and competitively block binding of Cyt c to Apaf-1, thereby inhibiting oligomerization of Apaf-1 [26,29]. Competition of Hsp90 and Cyt c for binding to Apaf-1 provides a mechanistic explanation for why cells overexpressing Hsp90 are highly resistant to Cyt c-mediated activation of caspase cascade. Therefore, we asked whether the NS1 expression affects the interaction of Hsp90 and Apaf-1 and thereby to induce apoptosis. Our results demonstrated that the interaction between NS1 and Hsp90 could competitively decrease the association of Apaf-1 and Hsp90 but increase the association of Apaf-1 and Cyt c. Based on the results we propose a model for the NS1-induced apoptosis (Figure 6): the binding of NS1 and Hsp90 might compete off the Apaf-1, resulting in the oligomerization of Apaf-1 which by turn promotes the interaction between Apaf-1 and Cyt c, and lead to the activation of caspase 9 and caspase 3 and cell apoptosis occur. Although many works are needed to prove the present model, our data might reveal a novel pathway for the NS1-mediated caspase cascade during the apoptosis induced by influenza A virus.

The outbreak of current new strain of influenza A virus subtype H1N1 and the epidemic of highly pathogenic avian influenza A virus H5N1 in the past years highlight that the influenza A viruses are globally important human and animal respiratory pathogens, and their detailed characterization is needed to facilitate our understanding for the disease pathogenesis and to help developing the vaccines and therapeutics. One can predict that any inhibitors that prevent the NS1-mediated apoptosis pathway would reduce disease severity and improve clinical outcomes.

## Competing interests

The authors declare that they have no competing interests.

## Authors' contributions

CFZ, YTY and XWZ mainly carried out construction of expression plasmids, isolation of NS1-binding partners by CytoTrap two-hybrid, mammalian two-hybrid, co-immunoprecitation assays, and wrote the manuscript. ZXY contributed to viruses culture. XLL contributed to electron microscopic analysis. ZLC contributed to flow cytometric analysis. HBS, YXH and PTH conceived the studies and participated in experimental design and coordination. All authors read and approved the final manuscript.
